# Flare on [^18^F]PSMA-1007 PET/CT after short-term androgen deprivation therapy and its correlation to FDG uptake: possible marker of tumor aggressiveness in treatment-naïve metastatic prostate cancer patients

**DOI:** 10.1007/s00259-022-05970-y

**Published:** 2022-09-26

**Authors:** Simona Malaspina, Otto Ettala, Tuula Tolvanen, Johan Rajander, Olli Eskola, Peter. J. Boström, Jukka Kemppainen

**Affiliations:** 1 Turku PET Centre, University of Turku and Turku University Hospital, Turku, Finland; 2 Department of Clinical Physiology and Nuclear Medicine, University of Turku and Turku University Hospital, Turku, Finland; 3 Department of Urology, University of Turku and Turku University Hospital, Turku, Finland; 4 Department of Medical Physics and Turku PET Centre, University of Turku and Turku University Hospital, Turku, Finland; 5 Turku PET Centre, Accelerator Laboratory, Åbo Akademi University, Turku, Finland

**Keywords:** Prostate cancer, PSMA, PET, [^18^F]PSMA-1007 PET/CT, ADT, Androgen deprivation therapy

## Abstract

**Purpose:**

Short-term androgen deprivation therapy (ADT) is known to increase heterogeneously prostate-specific membrane antigen (PSMA) expression. This phenomenon might indicate the potential of cancer lesions to respond to ADT. In this prospective study, we evaluated the flare on [^18^F]PSMA-1007 PET/CT after ADT in metastatic prostate cancer (PCa). Given that aggressive PCa tends to display FDG uptake, we particularly investigated whether the changes in PSMA uptake might correlate with glucose metabolism.

**Methods:**

Twenty-five men with newly diagnosed treatment-naïve metastatic PCa were enrolled in this prospective registered clinical trial. All the patients underwent [^18^F]PSMA-1007 PET/CT immediately before and 3–4 weeks after ADT initiation (degarelix). Before ADT, [^18^F]FDG PET/CT was also performed. Standardized uptake values (SUV)max of primary and metastatic lesions were calculated in all PET scans. Serum PSA and testosterone blood samples were collected before the two PSMA PET scans. The changes in PSMA uptake after ADT were represented as ΔSUVmax.

**Results:**

All the patients reached castration levels of testosterone at the time of the second [^18^F]PSMA-1007 PET/CT. Overall, 57 prostate, 314 lymph nodes (LN), and 406 bone lesions were analyzed. After ADT, 104 (26%) bone, 33 (11%) LN, and 6 (11%) prostate lesions showed an increase (≥ 20%) in PSMA uptake, with a median ΔSUVmax of + 50%, + 60%, and + 45%, respectively. Among the lesions detected at the baseline [^18^F]PSMA-1007 PET/CT, 63% bone and 46% LN were FDG-positive. In these metastases, a negative correlation was observed between the PSMA ΔSUVmax and FDG SUVmax (*p* < 0.0001). Moreover, a negative correlation between the ΔSUVmax and the decrease in serum PSA after ADT was noted (*p* < 0.0001).

**Conclusions:**

A heterogeneous increase in PSMA uptake after ADT was detected, most evidently in bone metastases. We observed a negative correlation between the PSMA flare and the intensity of glucose uptake as well as the decrease of serum PSA, suggesting that lesions presenting with such flare might potentially be less aggressive.

**Trial registration:**

NCT03876912, registered 15 March 2019.

**Supplementary Information:**

The online version contains supplementary material available at 10.1007/s00259-022-05970-y.

## Introduction

Androgen deprivation therapy (ADT) by surgical castration or administration of luteinising-hormone-releasing-hormone (LHRH) analogues is considered the first-line treatment in metastatic hormone-sensitive prostate cancer (PCa) [1, 2]. Despite favorable response rates, the onset of castration-resistance (CRPC) in patients with metastases is typical and could occur even within 1–2 years from the initiation of ADT [3, 4].

Therefore, defining the extent of the disease and the best treatment approach in metastatic PCa is paramount, in order to delay its progression towards CRPC and, possibly, prolong patients’ survival. Prostate-specific membrane antigen (PSMA) PET has gained increasing acceptance in the imaging of PCa, including its recent usage in primary staging setting [5–7]. PSMA is a type II membrane glycoprotein that is overexpressed in the prostate compared to other tissues, and its expression is tenfold higher in PCa cells compared to healthy prostate [8].

Preclinical studies on cell lines and animal models have demonstrated that short-term ADT upregulates the expression of PSMA in PCa cells in both hormone-sensitive and castration-resistant states [9, 10]. This phenomenon has been successively investigated in a few prospective clinical studies using PSMA PET imaging on PCa patients [11–13]. A heterogeneous increase in PSMA uptake, known as the PSMA flare, was observed in PCa lesions after short-term ADT, with considerable inter- and intrapatient variability.

In our previous prospective clinical trial on a cohort of 9 treatment-naïve PCa patients, we demonstrated a heterogeneous increase in PSMA uptake after 3–4 weeks of ADT, most evidently seen in bone lesions, followed mainly by a decrease of the uptake at 6–8 weeks after the initiation of the treatment [13]. Based on these preliminary results, we hypothesized that lesions exhibiting the flare in PSMA uptake might have a distinctive potential to response to ADT. However, research on this phenomenon is still limited, and the underlying biology of the PSMA flare remains uncertain and needs further clarification. Moreover, there is no consensus on the interpretation and the possible clinical significance of the flare on PSMA PET imaging.

The current prospective clinical trial represents a continuation of our previous study [13], and its aim is to assess the PSMA flare phenomenon after short-term ADT in a larger cohort of treatment-naïve metastatic PCa patients. In particular, since aggressive PCa tends to display FDG uptake [14], we investigated whether the changes in PSMA uptake after ADT might correlate with glucose metabolism.

## Materials and methods

### Subjects and study design

Men with newly diagnosed treatment-naïve metastatic PCa were enrolled in this prospective registered clinical trial. The inclusion criteria were (1) histologically confirmed adenocarcinoma of the prostate without neuroendocrine differentiation, small cell, or ductal features; (2) absence of previous surgical, radiation, or hormonal treatment of PCa; and (3) clinical stage Tany Nany M1. The exclusion criteria included (1) presence of uncontrolled serious infection and (2) presence or history of malignances other than PCa.

The metastatic status of the patients was defined as a quantifiable number of metastases documented by standard-of-care imaging (CT and/or bone scintigraphy) performed within 2 weeks from enrolment. All the patients underwent [^18^F]PSMA-1007 PET/CT immediately before and 3–4 weeks after the subcutaneous administration of ADT (degarelix, 240 mg). This time point was chosen as the most suitable to assess the flare in PSMA uptake according to the results of our previous study [13]. Before the initiation of ADT, a [^18^F]FDG PET/CT scan was performed during the same week of the baseline [^18^F]PSMA-1007 PET/CT to evaluate the aggressiveness of the PSMA-positive lesions. In addition, serum PSA and testosterone blood samples were collected before the two [^18^F]PSMA-1007 PET/CT scans. The change in serum PSA levels between these two time points was defined as ΔPSA.

### Imaging protocol

All PET/CT scans were carried out using a GE Discovery MI PET/CT scanner (GE Healthcare, Milwaukee, WI, USA). Fasting for 6 h was required before administration of [^18^F]FDG. The patients underwent whole-body PET/CT scans from mid-thigh to the vertex starting at 60 min and 50 min after receiving intravenous injection of [^18^F]PSMA-1007 and [^18^F]FDG, respectively. Low-dose CT scans were acquired for attenuation correction and anatomic correlation. The CT acquisition parameters were as follows: tube potential of 120 kV, tube current modulated between 10 and 120 mA, and noise index of 30. The PET scans were acquired in 3-dimensional mode with 2 min/bed positions. The sinogram data were corrected for deadtime, decay, and photon attenuation and, then, reconstructed on a 256 × 256 matrix. Image reconstruction utilized a Bayesian penalized-likelihood iterative reconstruction algorithm (QClear) with a β value of 500 for [^18^F]PSMA-1007 and 350 for [^18^F]FDG, incorporating random and scatter corrections.

### Radiopharmaceutical preparation

The synthesis of [^18^F]PSMA-1007 and [^18^F]FDG solutions for injection was conducted on-site at the Radiopharmaceutical Chemistry Laboratory of Turku PET Centre. [^18^F]FDG was synthesized following an analogous procedure as described previously [15]. A FASTLab® synthesizer (GE Healthcare, Waukesha, WI, USA) and FDG-phosphate cassettes were used for the production. The radiochemical purity exceeded 98%. [^18^F]PSMA-1007 was synthesized with TRASIS AllInOne synthesizer (TRASIS Radiopharma, Ans, Belgium) using the single-use cassettes supplied by TRASIS and reagents supplied by ABX (ABX advanced biochemical compounds Gmb, Radeberg, Germany). The radiochemical purity exceeded 93%.

### Imaging analysis and interpretation

Image analysis was performed using an AW 4.5 workstation (General Electrics (GE) Healthcare). An experienced nuclear medicine physician (S.M.) reviewed all the PET scans.

The PSMA PET findings were reported according to the current suggested procedure guidelines, taking the normal biodistribution and the possible pitfalls of the tracers into consideration [16]. All the metastases documented on conventional imaging (CT and bone scintigraphy) that showed PSMA-uptake at the baseline [^18^F]PSMA-1007 PET/CT were included in the analysis. Moreover, lesions were considered malignant on PSMA PET imaging if the following criteria were met: [^18^F]PSMA-1007 focal uptake above the local background in the prostate for T-staging; [^18^F]PSMA-1007 uptake above the blood pool with corresponding CT finding (also normally sized lymph nodes) in a site typical for prostate cancer for N-staging; [^18^F]PSMA-1007 uptake above the blood pool with corresponding CT finding (e.g., sclerotic or lytic bone lesion) in a site typical for prostate cancer for M-staging.

All the PSMA-positive lesions were then carefully matched with [^18^F]FDG PET/CT. Lesions with tracer uptake above the blood pool were considered FDG-positive.

The standardized uptake value (SUV)max of the PSMA-avid lesions in the prostate and in PCa metastases was calculated. The changes in PSMA uptake after ADT were represented as ΔSUVmax. The PSMA-positive lesions were then divided into two groups. The first group included all the lesions with a ΔSUVmax ≥  + 20% (PSMA flare) while the second group consisted of the remaining lesions exhibiting either a decrease or no change (< 20%) of SUVmax. The FDG SUVmax was further divided into three categories: ≤ blood pool (FDG-negative), > blood pool up to 10 (mild to moderate uptake), and > 10 (strong uptake). All data were collected using a RedCap database [17].

### Statistical analysis

The descriptive data are presented as a median value, interquartile range (IQR), and range. Pearson’s coefficient was used to assess the correlation between PSMA SUVmax and FDG SUVmax as well as the correlation between PSMA ΔSUVmax, FDG SUVmax, and ΔPSA. Welch’s analysis of variance (Anova) was used to compare PSMA ΔSUVmax to different classes of FDG SUVmax. *p* values < 0.05 were considered statistically significant. Statistical analysis was carried out using JMP® pro 16 software.

## Results

Twenty-five patients were prospectively enrolled in the study and completed all the three PET scans. The patients’ characteristics are presented in Table 1. Their median age was 74 years old (IQR 70–78; range 63–84), and the median PSA before the initiation of ADT was 49 ng/ml (IQR 33–140; range 15–5000). The median time interval from the baseline [^18^F]PSMA-1007 PET/CT scan to the administration of ADT was 2 days (IQR 1–3; range 0–8). The median time interval between the baseline [^18^F]PSMA-1007 PET/CT and the [^18^F]FDG scan was 1 day (IQR 1–2; range 1–6). The second [^18^F]PSMA-1007 PET/CT scan was performed after a median of 27 days (IQR 21–30; range 20–33) from ADT initiation. The median administered activity of [^18^F]PSMA-1007 and [^18^F]FDG were 255 MBq (IQR 251–259; range 241–278) and 368 MBq (IQR 333–381; range 278–398), respectively. Scanning time after the radiotracer injection was 60 min (median, IQR 59–60) for [^18^F]PSMA-1007 and 50 min (median, IQR 50–50) for [^18^F]FDG. All the patients reached castration levels (testo < 1.7 nmol/L) within the time of the second [^18^F]PSMA-1007 PET/CT scan. Serum PSA decreased in all the patients at 3–4 weeks after ADT, with a median decrease of 87% (IQR 81–92; range 32–99).

**Table 1:** Patients’ demographics

Age	Median (IQR; range)
Years	74 (70–78; 63–84)
PSA at baseline	Median (IQR; range)
ng/ml	49 (33–140; 15–5000)
S-testo at baseline	Median (IQR; range)
nmol/L	12 (7–17; 2–27)
Biopsy GGG^a^	*n* (%)
1	0 (0)
2	0 (0)
3	3 (12)
4	4 (16)
5	18 (72)
Clinical T-category	*n* (%)
cT1	0 (0)
cT2	2 (8)
cT3	19 (76)
cT4	4 (16)

^a^Gleason grade group

All the patients presented with strong PSMA uptake in the prostate gland at the baseline, 10 of whom presented uptakes that extended to the seminal vesicles. All the patients had PSMA-avid lymph node (LN) metastases in the pelvis, while 12 of them also had pathological uptakes in retroperitoneal and/or mediastinal LNs. Twenty-three patients presented with PSMA-positive bone lesions. Finally, two patients had PSMA-avid lung nodules. An overview of the positive lesions detected at the baseline [^18^F]PSMA-1007 PET/CT for each patient is depicted in Supplemental Fig. 1.

In all the men, an increase of PSMA uptake after ADT was observed in at least one bone and/or lymph node metastasis (Supplemental Fig. 2). In particular, except for one man, all patients with bone metastases (*n* = 22) exhibited flare of PSMA uptake in the bone at the second PSMA PET scan, despite significant inter-patient variability (Supplemental Fig. 2). A total of 57 prostate/seminal vesicle, 314 LN (250 regional and 64 extra-regional), 406 bone and 5 lung lesions that were positive on the baseline [^18^F]PSMA-1007 PET/CT were included in the analysis. The changes observed in the PSMA uptake after ADT at the lesion level are represented in Table 2. An increase in PSMA uptake was observed in 26% of the bone lesions, with a median ∆SUVmax of + 50% (IQR 32–72; range 20–161). This flare in PSMA uptake was more evident in the bone metastases compared to the LN or prostate, where 11% and 11% of the lesions showed a median PSMA increase (∆SUVmax) of + 60% (IQR 32–114; range 20–222) and +45% (IQR 20–106; range 23–134), respectively. The lung nodules did not show any flare in PSMA uptake. The remaining lesions showed either a decrease or no change in PSMA uptake (Table 2). No lesion became PSMA-negative (uptake below the blood pool) at the second PSMA PET scan.

**Table 2:** Changes in PSMA uptake after ADT

Lesion type	Lesions (*n*) at baseline PSMA PET	Increase (≥ 20%) of PSMA uptake, *n* (%)	∆SUVmax%, median (IQR; range)	No change/decrease (< 20%) of PSMA uptake, *n* (%)	∆SUVmax% median (IQR; range)
Prostate	57	6 (11%)	+ 45% (20–106; 23–134)	49 (89%)	− 28% (− 45 to − 7; − 78 to 14)
Lymph nodes	314	33 (11%)	+ 60% (32–114; 20–222)	281 (89%)	− 51% (− 74 to − 21; − 99 to 9)
Bone	406	104 (26%)	+ 50% (32–72; 20–161)	302 (74%)	− 33% (− 53 to − 15; − 90 to 14)
Visceral (lung)	5	-	-	5 (100%)	− 40% (− 49 to − 22; − 53 to − 16)

**Fig. 1:**
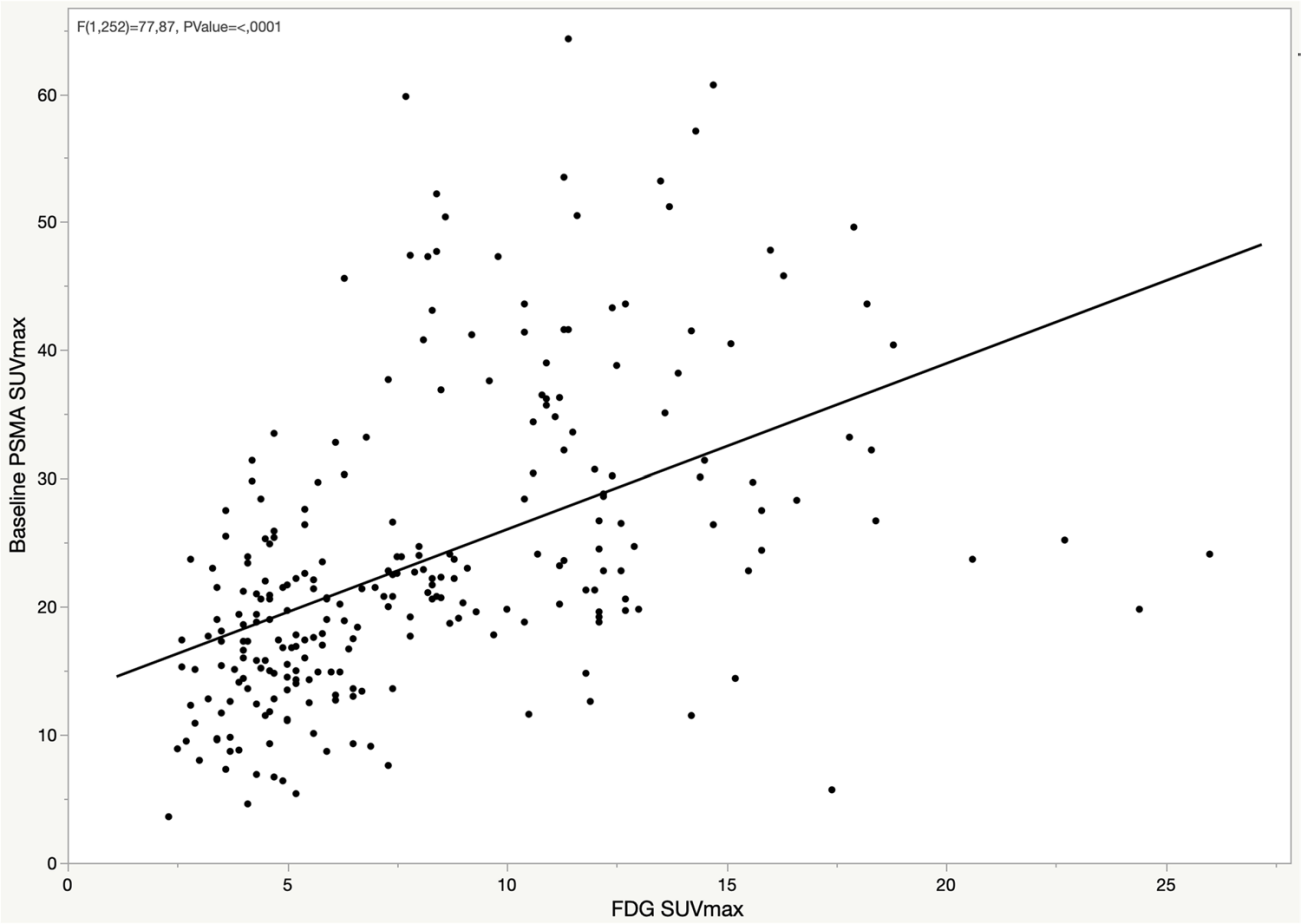
Correlation between baseline PSMA SUVmax and FDG SUVmax in bone lesions

**Fig. 2:**
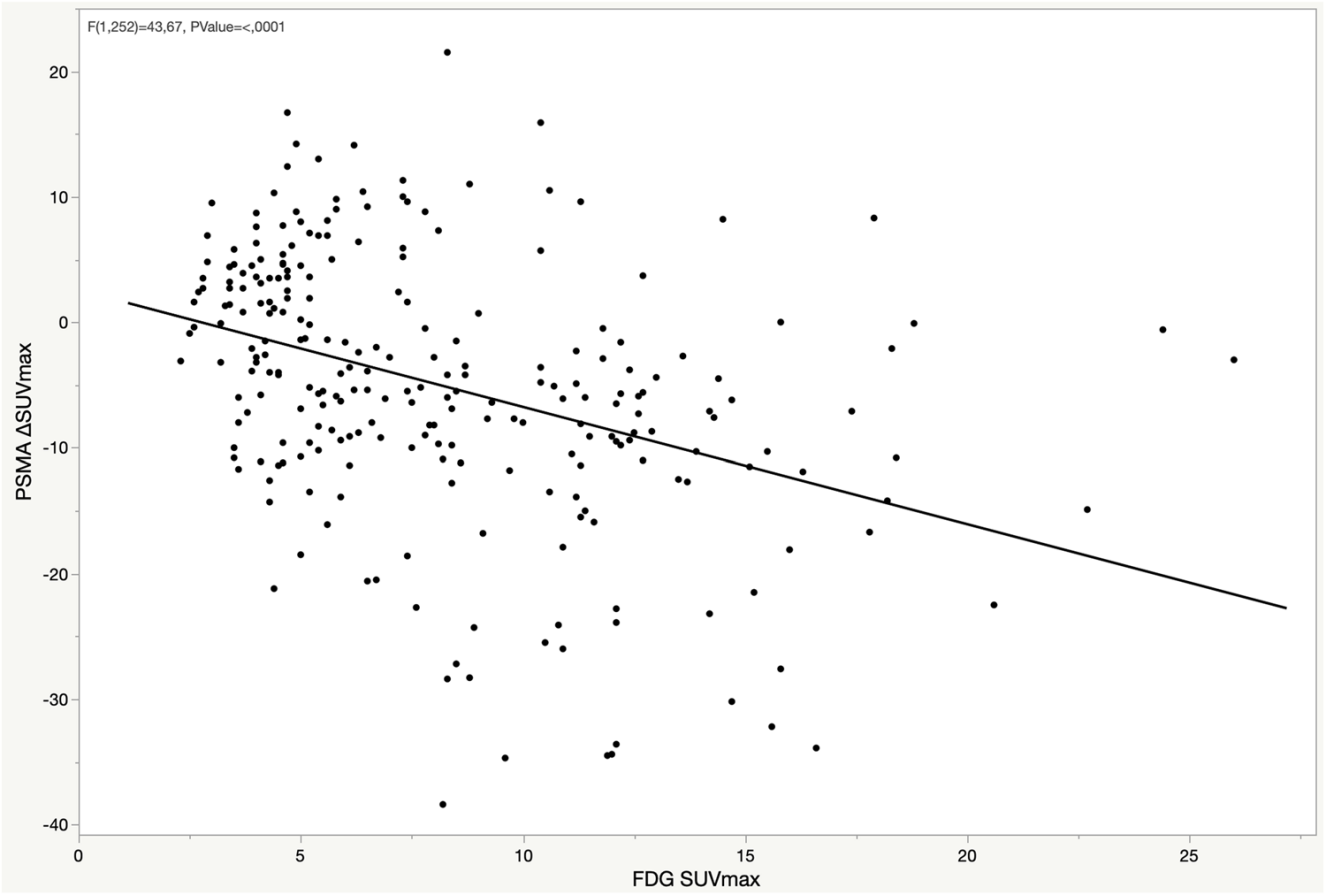
Correlation between PSMA ΔSUVmax and FDG SUVmax in bone lesions

**Fig. 3:**
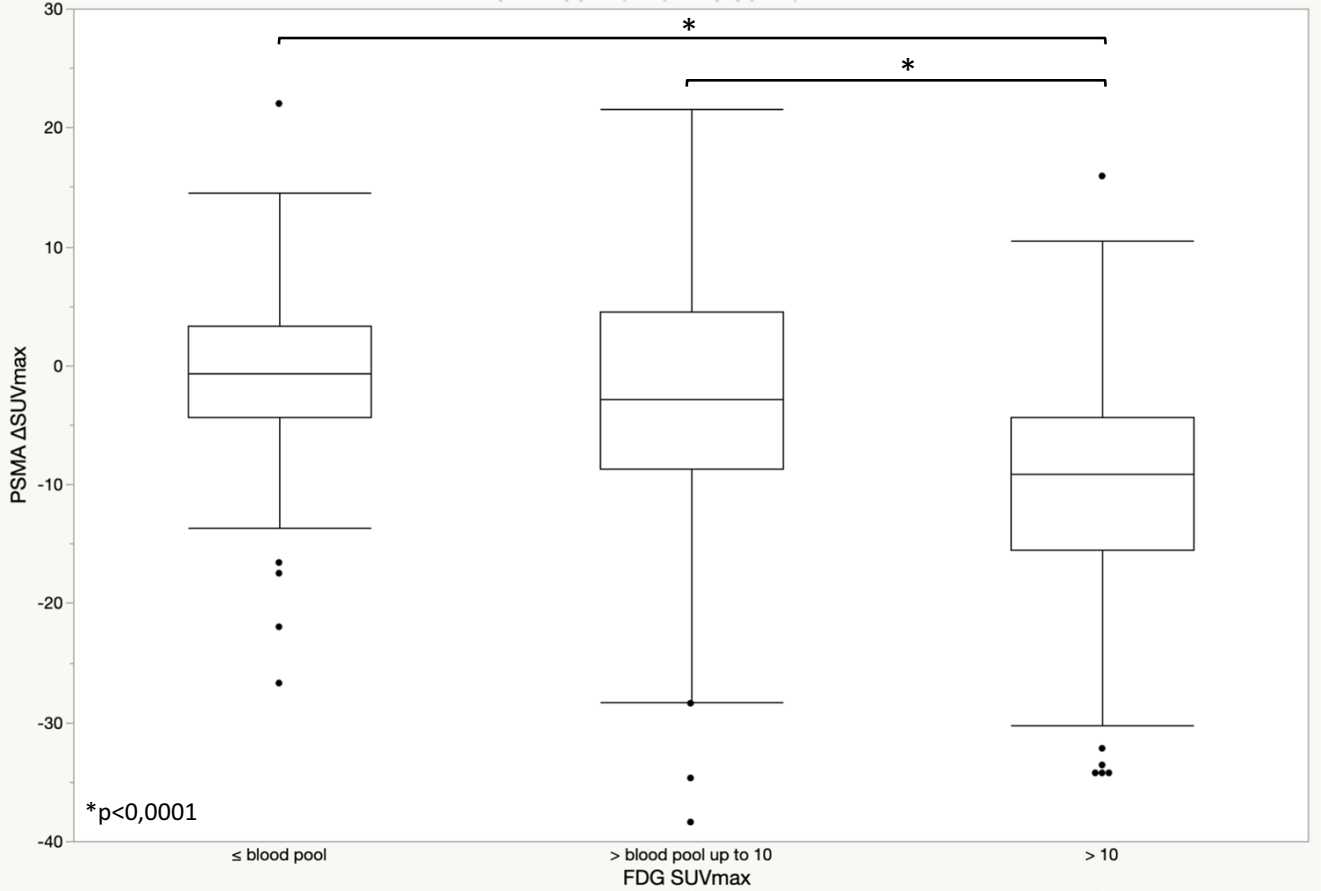
Box plot for PSMA ΔSUVmax according to FDG SUVmax in bone lesions

**Fig. 4:**
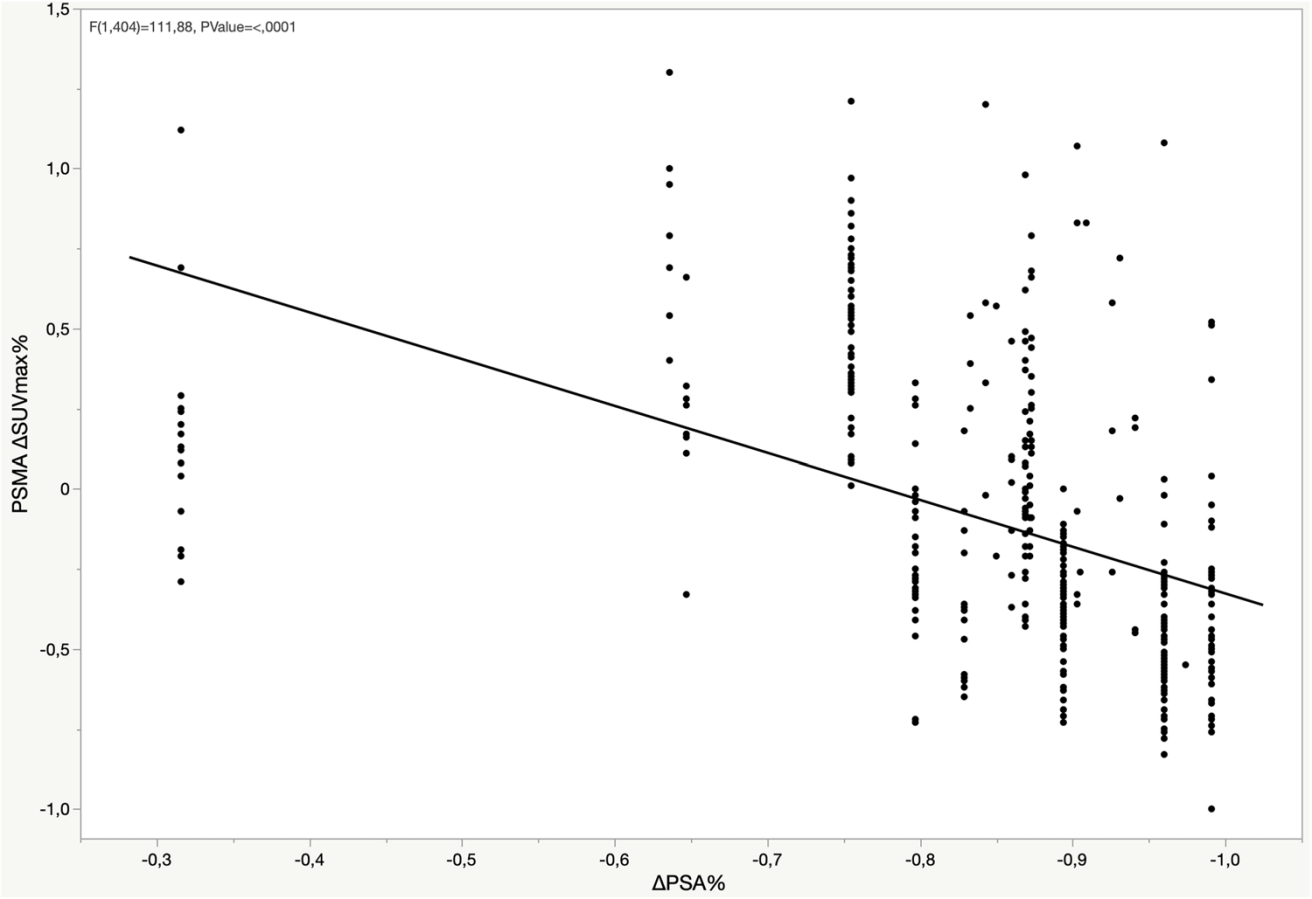
Correlation between PSMA ΔSUVmax and ΔPSA in bone lesions

At the baseline, among the PSMA-positive lesions, 27 (47%) prostate, 144 (46%) LN, 254 (63%) bone, and 3 (60%) lung lesions were positive on the [^18^F]FDG PET/CT. On comparing the intensity of baseline PSMA SUVmax and FDG SUVmax in the bone metastases, a positive correlation was observed (*p* < 0.001) (Fig. 1). Moreover, the comparison of the changes in PSMA uptake after ADT (∆SUVmax) and the FDG SUVmax showed a negative correlation (*p* < 0.001), indicating that lesions presenting the PSMA flare had less intense FDG uptake (Fig. 2). Similar correlations were observed in the lymph nodes (Supplemental Figs. 3–4), while correlations in the prostate lesions were not significant (Supplemental Figs. 5–6). The comparison between PSMA ∆SUVmax and FDG uptake, including the FDG-negative lesions (SUVmax < blood pool), is depicted in Fig. 3. A significant difference in the PSMA ∆SUVmax was observed between the bone metastases with strong FDG uptake (SUVmax > 10) and those having either a moderate FDG uptake or uptake ≤ blood pool (*p* < 0.001). Similar results were observed in the lymph nodes, but not in the prostate lesions (Supplemental Figs. 7–8). The correlation between PSMA ∆SUVmax and changes in serum PSA (∆PSA) is depicted in Fig. 4. The results showed a negative correlation (*p* < 0.001), indicating that the PSMA flare phenomenon was less evident in patients experiencing a rapid decrease in serum PSA (Fig. 4).

**Fig. 5:**
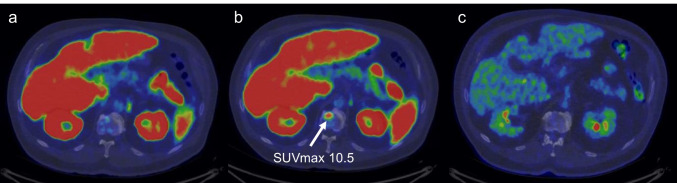
New PSMA bone uptake on L1 vertebra detected in the [^18^F] PSMA-1007 PET/CT after ADT. The uptake was not seen in the [^18^F]FDG PET/CT scan. **a** Baseline [^18^F] PSMA-1007 PET/CT. **b** [^18^F] PSMA-1007 PET/CT after ADT. **c** Baseline [^18^F]FDG PET/CT

Ten patients with known bone lesions presented with new PSMA bone uptakes at the second [^18^F]PSMA-1007 PET/CT scan (median SUVmax 5; IQR 5–10; range 4–18). An example is illustrated in Fig. 5. None of these uptakes was positive on the [^18^F]FDG PET/CT scan.

## Discussion

In this prospective trial, we demonstrated that short-term ADT increased PSMA uptake in metastatic treatment-naïve hormone-sensitive PCa. This PSMA flare was observed in all the patients, most evidently in bone metastases. According to the negative correlation between FDG uptake and changes of PSMA uptake after ADT, the flare phenomenon seems to be linked to less aggressive behavior of the lesions.

The possible association between the flare in PSMA uptake and glucose metabolism had not been investigated so far. It is well known that FDG uptake and its intensity is associated with more aggressive PCa [14, 18, 19]. Given the metastatic status of our patient cohort, the presence of FDG-positive lesions was expected. Interestingly, we observed a negative correlation between the flare in PSMA uptake and the intensity of FDG uptake in bone metastases and, to less extent, in lymph node metastases. This suggests that lesions presenting with the flare and milder FDG uptake might be less aggressive compared to lesions without the flare and with stronger FDG uptake. Therefore, this flare phenomenon might be able to identify more aggressive metastases, and potentially predict the response to ADT and the progression to CRPC.

Bone flare is a phenomenon that has already been observed in bone scintigraphy as either an increase in metabolic activity or the presence of new lesions within few weeks to few months of oncological treatments in PCa as well as other malignances. This phenomenon has been considered as a sign of favorable response to treatment [20]. In our study, 22/23 patients with bone metastases presented with flare of PSMA uptake after ADT in the bone. Moreover, ten of them exhibited new PSMA bone uptakes without anatomical correspondence at the second PSMA PET scan. Whether these new PSMA uptakes are true metastases or hormone-sensitive tissue reaction as part of the flare remains to be confirmed by longer follow-up. Nevertheless, these uptakes were negative also in the baseline FDG PET scan. This finding is consistent with our observation of the presence of PSMA flare in lesions with mild or no FDG-uptake, suggesting a less aggressive behavior. In view of this, the PSMA flare might resemble the flare observed in bone scintigraphy. The possible pathogenesis of the flare in bone tissue might be caused by the healing processes of new bone formation after short-term treatment. In addition, an immune response, specifically a T-cell reaction accompanied by the release of pro-inflammatory cytokines might possibly be involved in the flare phenomenon [20].

However, we demonstrated that the phenomenon is not bone-specific, as the increase in PSMA uptake was also observed in the lymph nodes and prostate lesions. It is possible that a similar immune response might be occurring in those tissues as well. Nevertheless, it is unlikely that this would be the only mechanism involved in the increase in PSMA uptake. A recent study on genomically characterized patient-derived xenografts (PDX) observed increases in PSMA and androgen receptor (AR) mRNA as well as tumor microdensity after ADT in castration-sensitive models [21]. Moreover, this study observed that the model exhibiting increased PSMA and AR mRNA had an intact PTEN gene, while the model with PTEN loss exhibited repressed AR transcriptional signaling. Given that the loss of PTEN gene is usually associated with more aggressive disease, these findings might corroborate our hypothesis of the PSMA flare as a marker of less aggressive disease. In view of this, understanding the possible molecular mechanisms and genetic phenotypes that modulate the heterogeneous PSMA responses to ADT might help to better understand the flare phenomenon.

The possible clinical significance of the PSMA flare still requires further clarification. Given the heterogeneity of the phenomenon, increase in the diagnostic performances is most likely limited, as no changes in staging were observed in our cohort of patients. On the other hand, identifying patients at risk of rapid progression would allow the implementation of appropriate follow-up strategies or further therapies. In particular, knowing that certain metastases are potentially more prone to progress might help in selectively treat those with stereotactic radiotherapy. However, this might not always be feasible in high-volume disease. Moreover, the impact of targeted radiotherapy of metastatic lesions on patient outcome is still under debate. Another aspect to consider is whether the PSMA flare might have an impact on the planning of radionuclide therapies. A recent prospective pilot study demonstrated the safety and the feasibility of [^177^Lu]PSMA treatment in hormone-sensitive metastatic PCa [22]. In this scenario, hormone-sensitive patients who exhibit more evident PSMA flare after ADT could have increased binding sites for radionuclide therapy. Further prospective trials are needed to confirm whether ADT-related PSMA flare could improve the outcome of [^177^Lu]PSMA therapy.

In this study, FDG PET has been used to select potentially aggressive PCa lesions and, through that, to investigate the possible correlation between FDG uptake and the PSMA flare phenomenon. Based on the results of our study, we would not recommend the use of FDG PET in the clinical practice for primary staging of PCa. However, it would be scientifically interesting to observe whether FDG-positivity could have a predictive value in the development of castration resistance. Moreover, as we hypothesize about the PSMA-flare, FDG-positivity could also have a potential role in selecting aggressive lesions for metastases-targeted therapies. Longer follow-up will hopefully give insight into these aspects.

We observed that the decrease in serum PSA is correlated negatively with the presence of the PSMA flare; in other words, PSA decreased more rapidly in patients whose majority of lesions did not present with the PSMA flare. Serum PSA and several other PSA-related parameters, such as PSA at diagnosis, PSA nadir, time to nadir, and percentage of decrease, have been widely accepted as prognostic factors to predict response to therapy [23, 24]. One might expect that a rapid decrease in serum PSA after ADT would be the result of rapid PCa cell death and therefore would predict better response to therapy [23, 25]. However, interestingly, some studies have observed that a slower decrease in serum PSA, particularly meaning a longer time to nadir, is associated with better response and longer survival [26–29]. The mechanisms responsible for the association between a rapid decline in PSA and a worsening prognosis are still not clear. One explanation might be that the rapid fall of PSA is related to a transcriptional effect on PSA production rather that cell death. Another possibility is that a rapid removal of hormone-sensitive cells can initiate the growth of castration-resistant cells [27, 28]. Our results seem to be consistent with this concept, since the serum PSA decreased more rapidly in patients with potentially more aggressive lesions. If proven right, this might corroborate the hypothesis of the PSMA flare as a favorable prognostic factor. However, the follow-up time for serum PSA in our cohort is too short to draw conclusions and longer follow-up is needed to confirm this hypothesis.

Our results regarding the PSMA flare in hormone-sensitive patients are consistent with the previous prospective studies that investigated the effect of short-term ADT on PSMA uptake [10, 11, 13]. Emmet et al. reported a reduction of PSMA uptake after short-term ADT in 8 hormone-sensitive PCa patients, according to a patient-based analysis and a single median SUVmax value [12]. However, despite similar patient characteristics and imaging time-points, our study was performed on a larger cohort of patients and the image analysis was lesion-based. These considerations are likely to explain the differences in the reported findings.

The use of SUVmax as the only parameter for tracer uptake might be a limitation of this study. However, we used a 20% as a cut-off to define the increase in PSMA uptake in order to avoid any possible variation in the SUVmax due to technical reproducibility aspects. Moreover, all the patients were scanned with the same camera in order to minimize possible technical variation and no differences in injected activities or scanning times were observed. The presence of only one PET reader might also be considered as a limitation. However, the aim of this study was not to assess the diagnostic performance of [^18^F]PSMA-1007 PSMA PET, but rather to investigate the phenomenon of the flare in PSMA uptake in patients with already known distant metastases. Moreover, we had already assessed the inter-reader agreement in our previous trial [13] that did not demonstrated significant differences between the readers. Finally, the absence of histological verification of potential metastases, especially in the bone, might be another limitation, considering that non-specific bone uptakes might be encountered in [^18^F]PSMA-1007 PET imaging [6]. However, the presence of bone metastases was confirmed on conventional imaging performed within 2 weeks from enrolment. Moreover, to avoid the risk of possible false-positive bone uptakes, only those PSMA uptakes with corresponding finding on CT (sclerotic or lytic lesion) were included in our analysis.

## Conclusion

A heterogeneous flare in PSMA uptake after short-term ADT was observed in metastatic treatment-naïve PCa patients, most evidently in bone lesions. There seems to be a negative correlation between the PSMA flare and the intensity of FDG uptake, suggesting that lesions presenting with the flare might potentially be less aggressive. Moreover, serum PSA decreased less rapidly in patients with a higher number of lesions exhibiting the PSMA flare, which might also potentially be a sign of less aggressiveness. It is still unclear whether the flare phenomenon could predict a better response to ADT. Longer follow-up is needed to confirm these hypotheses. All the patients in the current trial will receive follow-up and will be scanned with [^18^F]PSMA-1007 PET/CT at an interval of 1 year and at onset of CRPC. The future results will hopefully provide further insight into this matter.

## Supplementary Information

Below is the link to the electronic supplementary material.

Supplementary file1 (PPTX 2787 KB)

## Data Availability

Data are available on reasonable request to the corresponding author.
