# Supplementary material for: Flare on [18F]PSMA-1007 PET/CT after short-term androgen deprivation therapy and its correlation to FDG uptake: possible marker of tumor aggressiveness in treatment-naïve metastatic prostate cancer patients
Source: Eur J Nucl Med Mol Imaging. 2022 Sep 26;50(2):613–21. doi: 10.1007/s00259-022-05970-y (PMC9816233; doi:10.1007/s00259-022-05970-y)
Supplement: Supplementary file 1 — Supplementary file1 (PPTX 2787 KB) [file 259_2022_5970_MOESM1_ESM.pptx]

## Slide 1
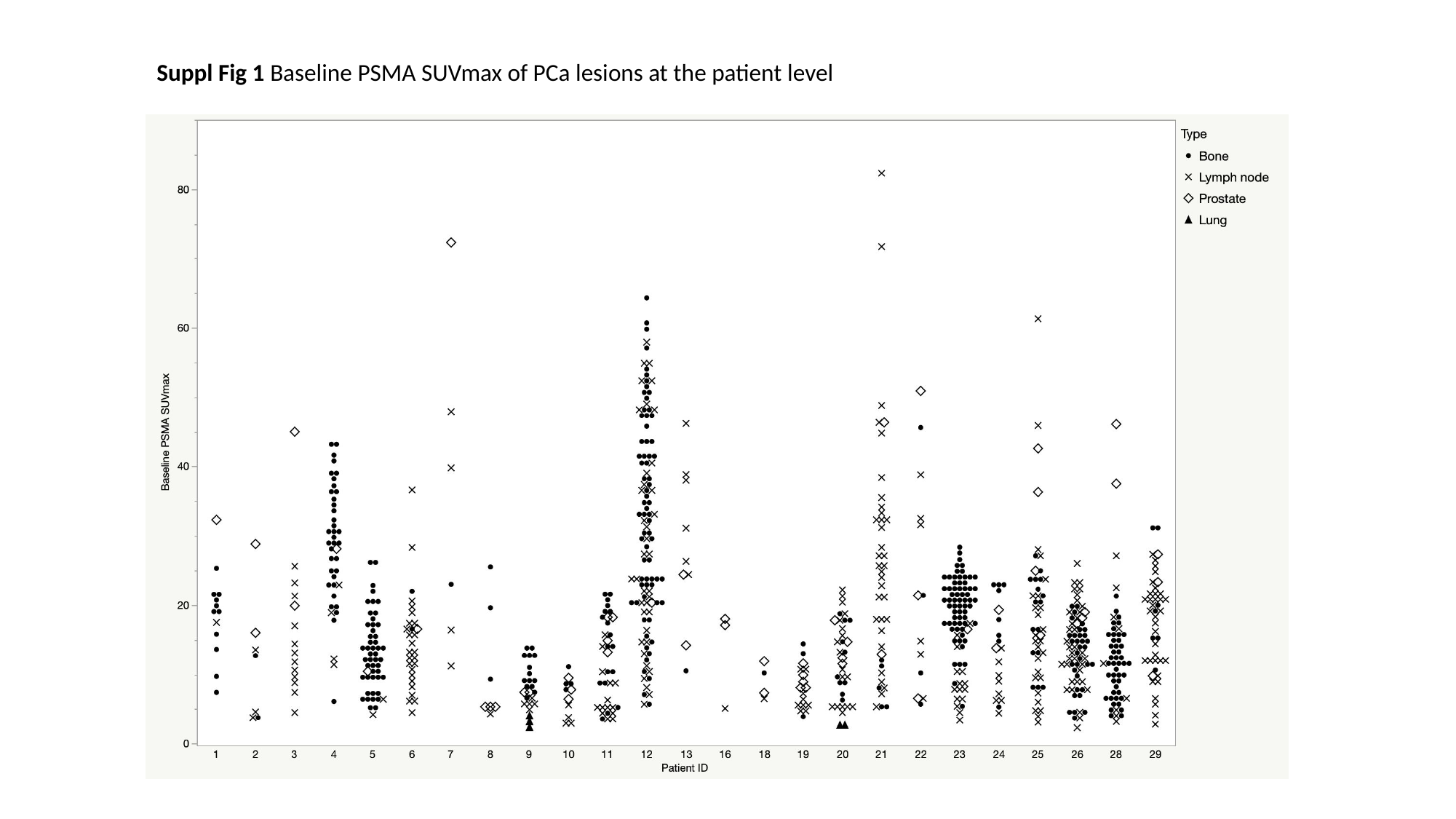

Suppl Fig 1 Baseline PSMA SUVmax of PCa lesions at the patient level

## Slide 2
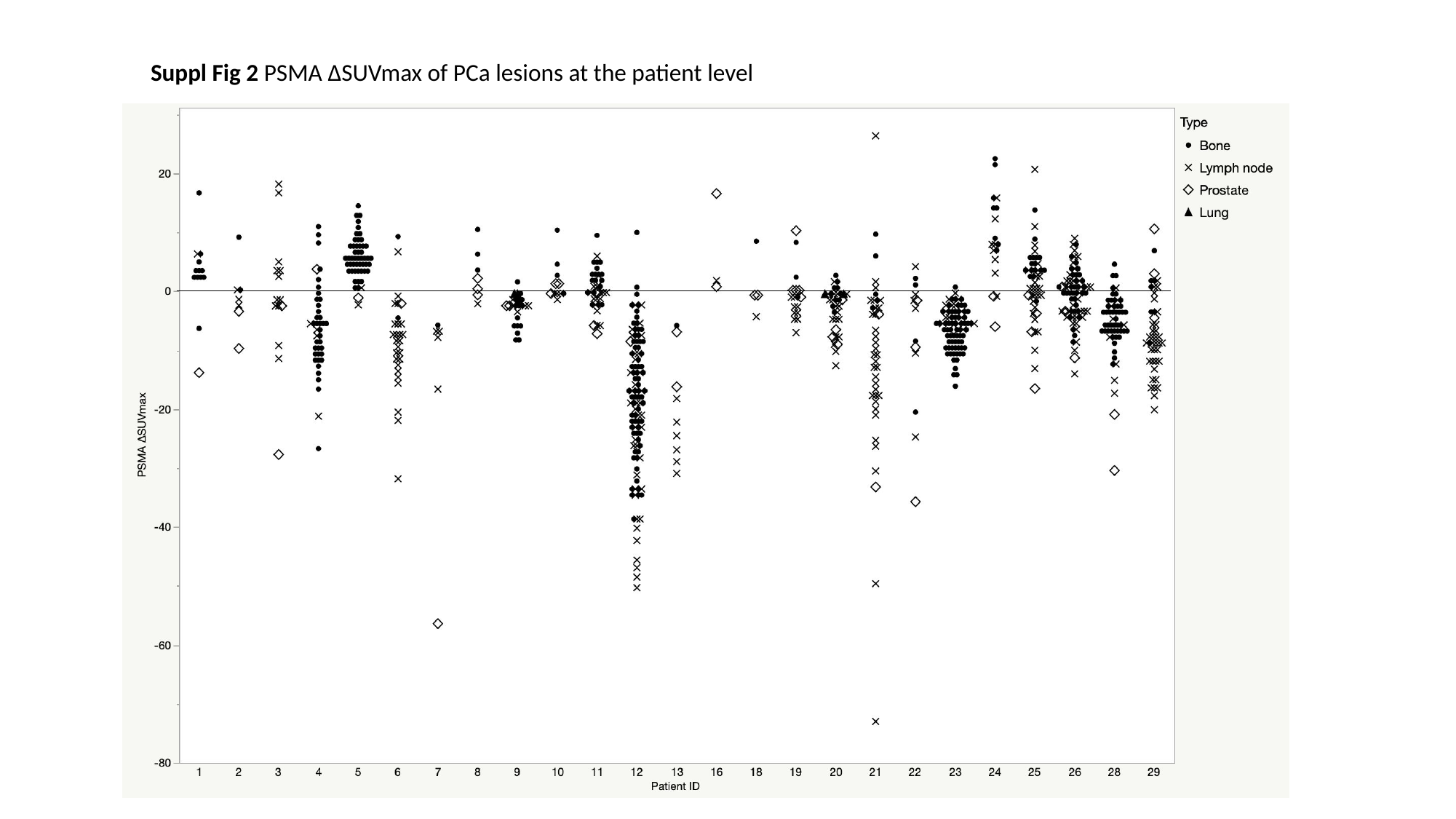

Suppl Fig 2 PSMA ΔSUVmax of PCa lesions at the patient level

## Slide 3
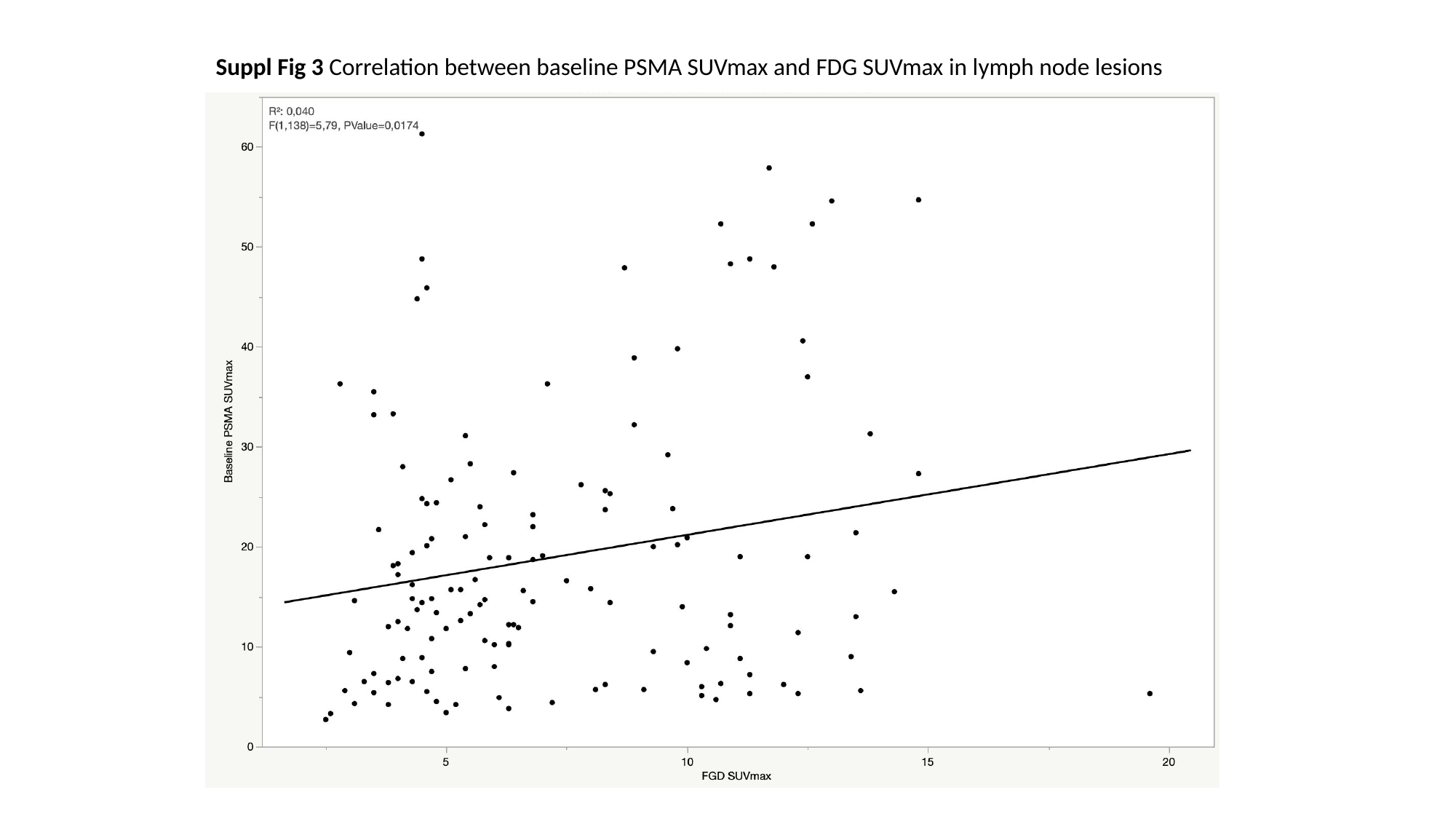

Suppl Fig 3 Correlation between baseline PSMA SUVmax and FDG SUVmax in lymph node lesions

## Slide 4
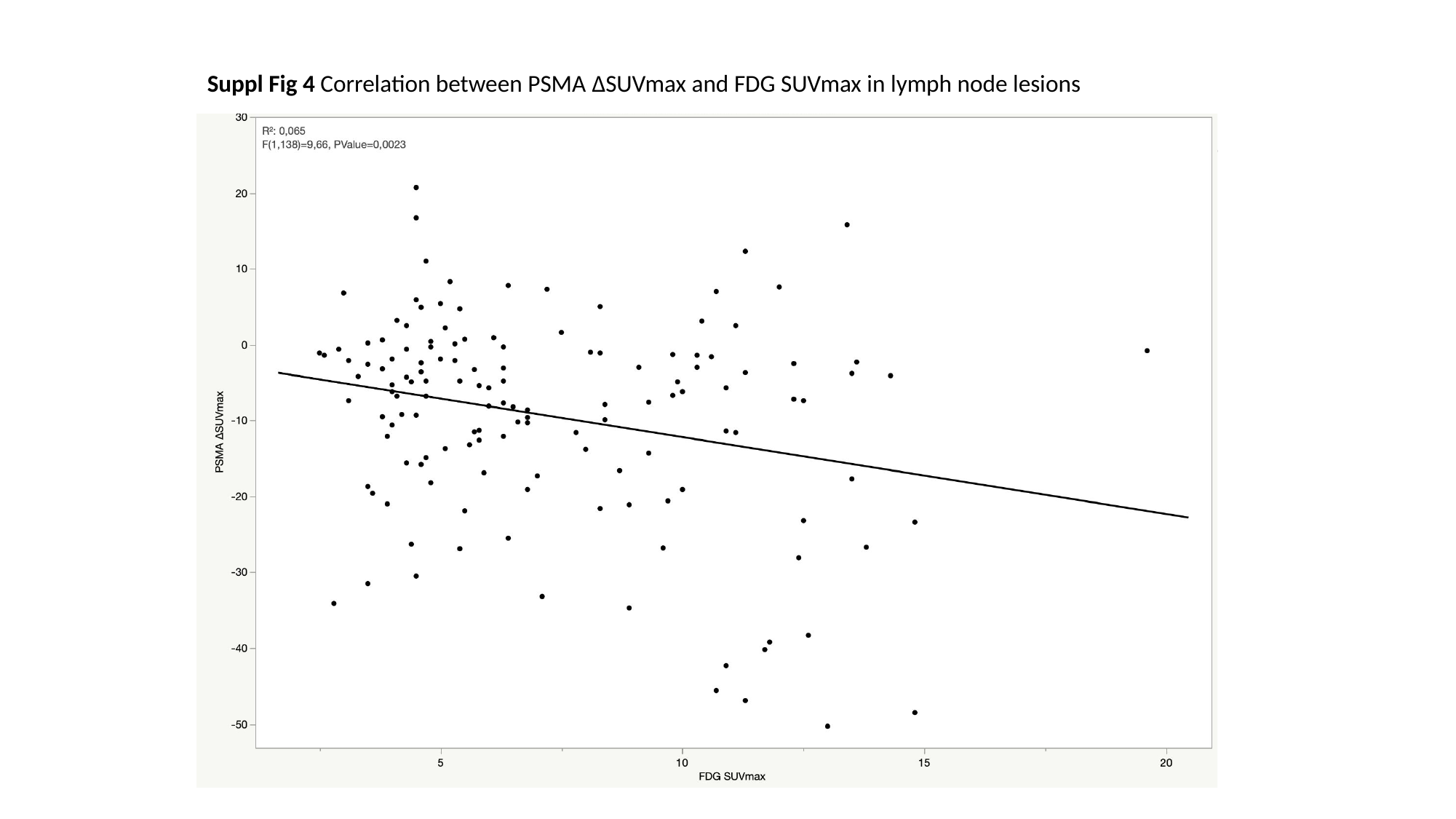

Suppl Fig 4 Correlation between PSMA ΔSUVmax and FDG SUVmax in lymph node lesions

## Slide 5
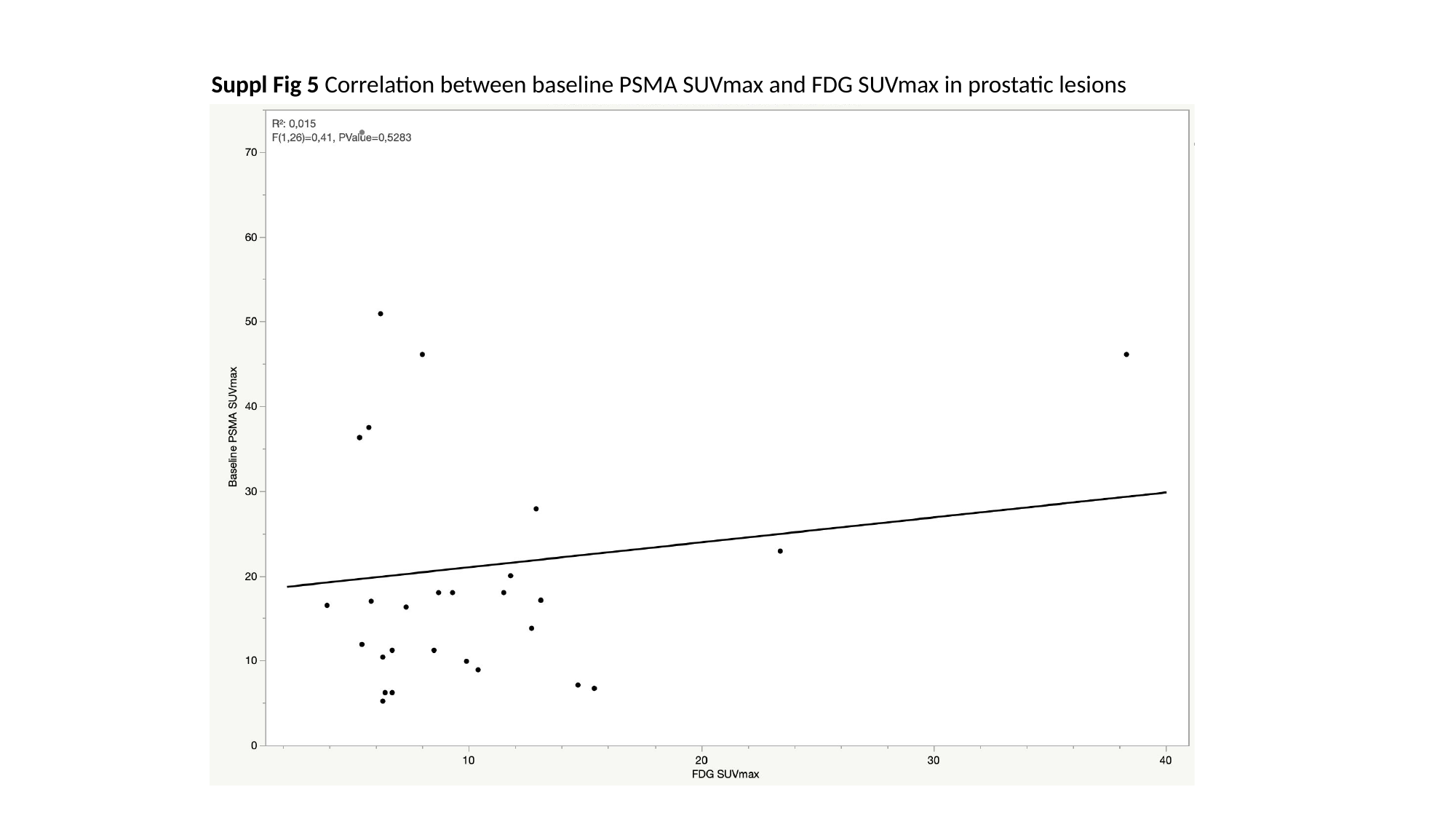

Suppl Fig 5 Correlation between baseline PSMA SUVmax and FDG SUVmax in prostatic lesions

## Slide 6
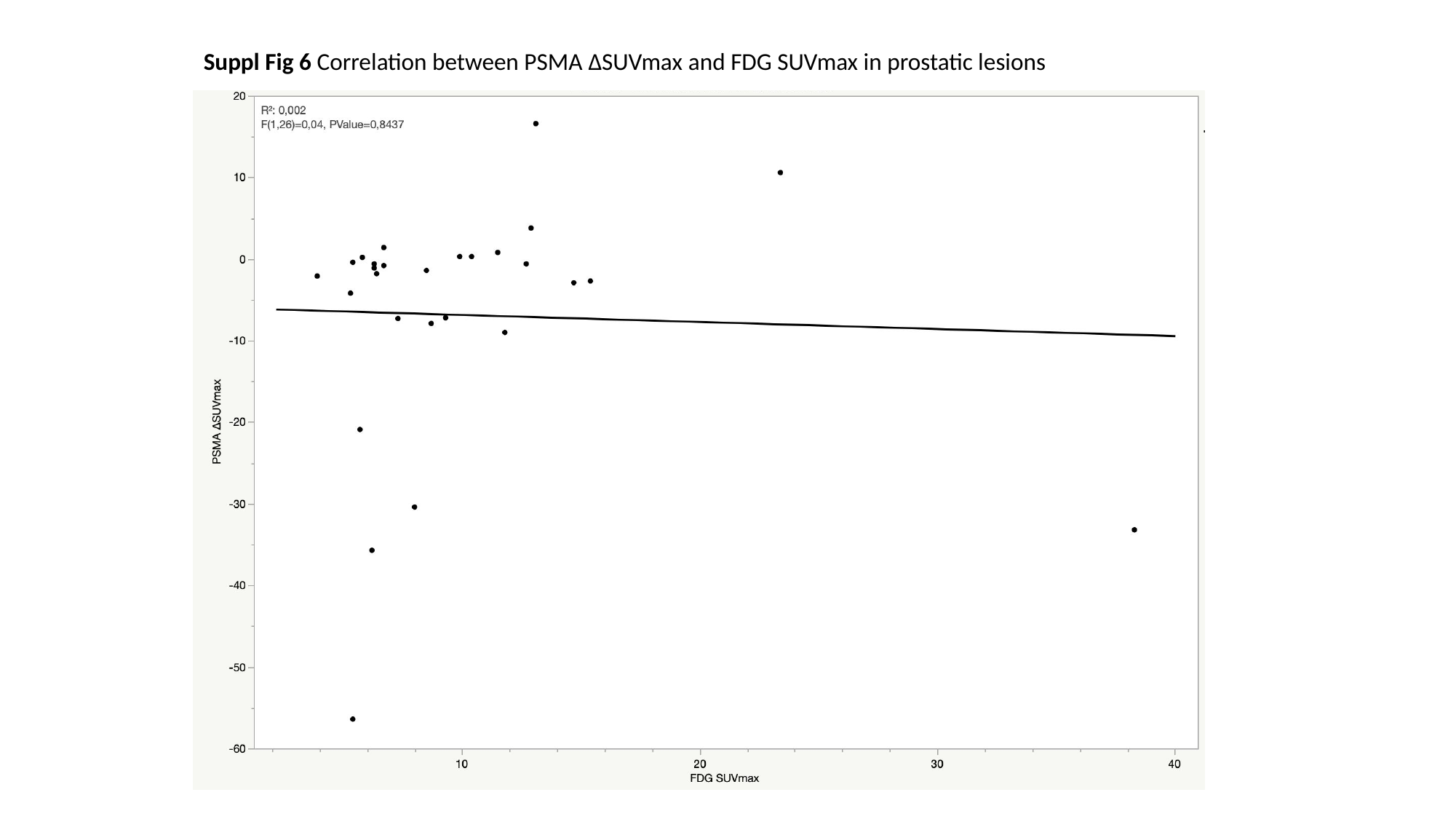

Suppl Fig 6 Correlation between PSMA ΔSUVmax and FDG SUVmax in prostatic lesions

## Slide 7
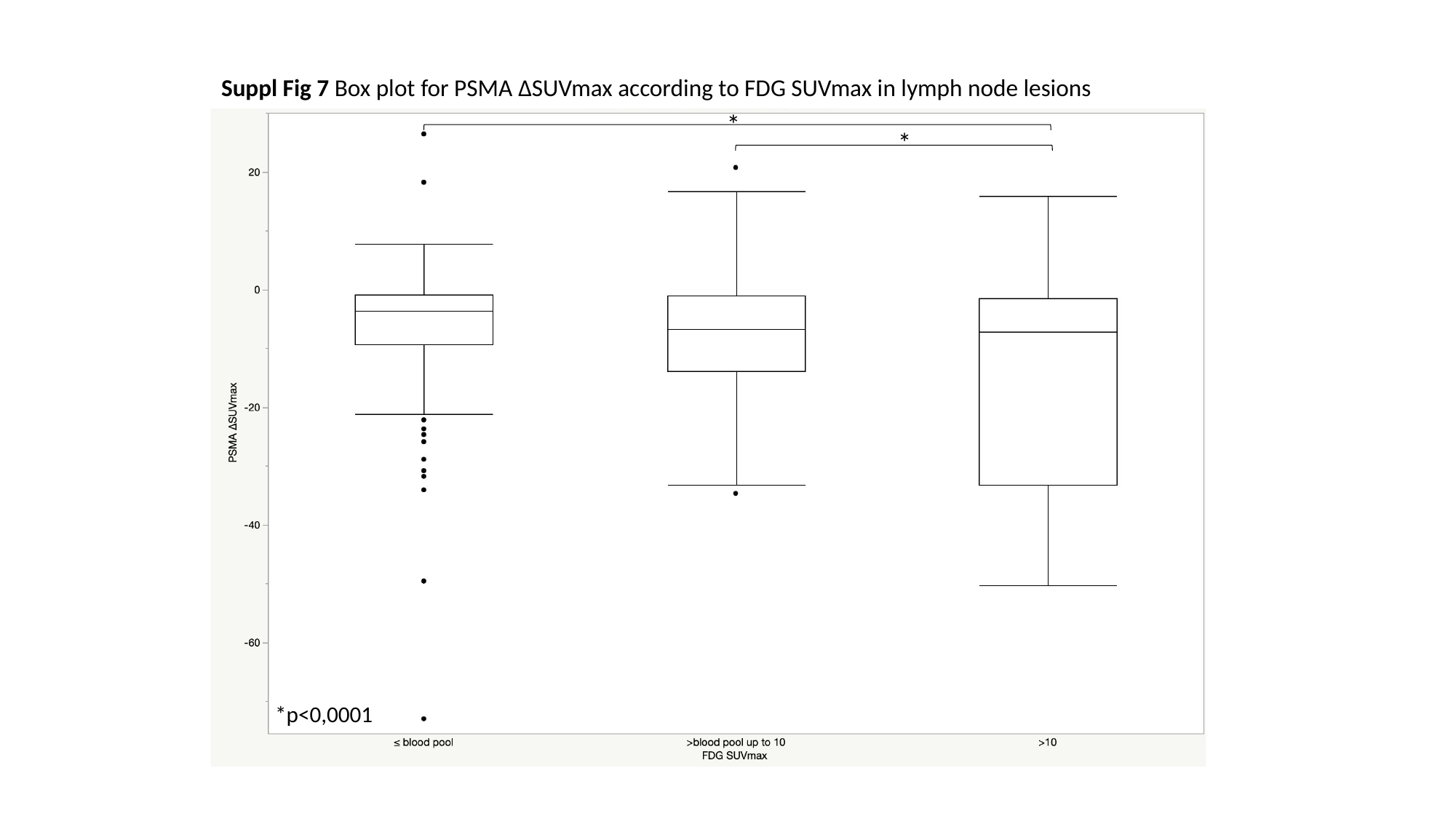

Suppl Fig 7 Box plot for PSMA ΔSUVmax according to FDG SUVmax in lymph node lesions
*
*
*p<0,0001

## Slide 8
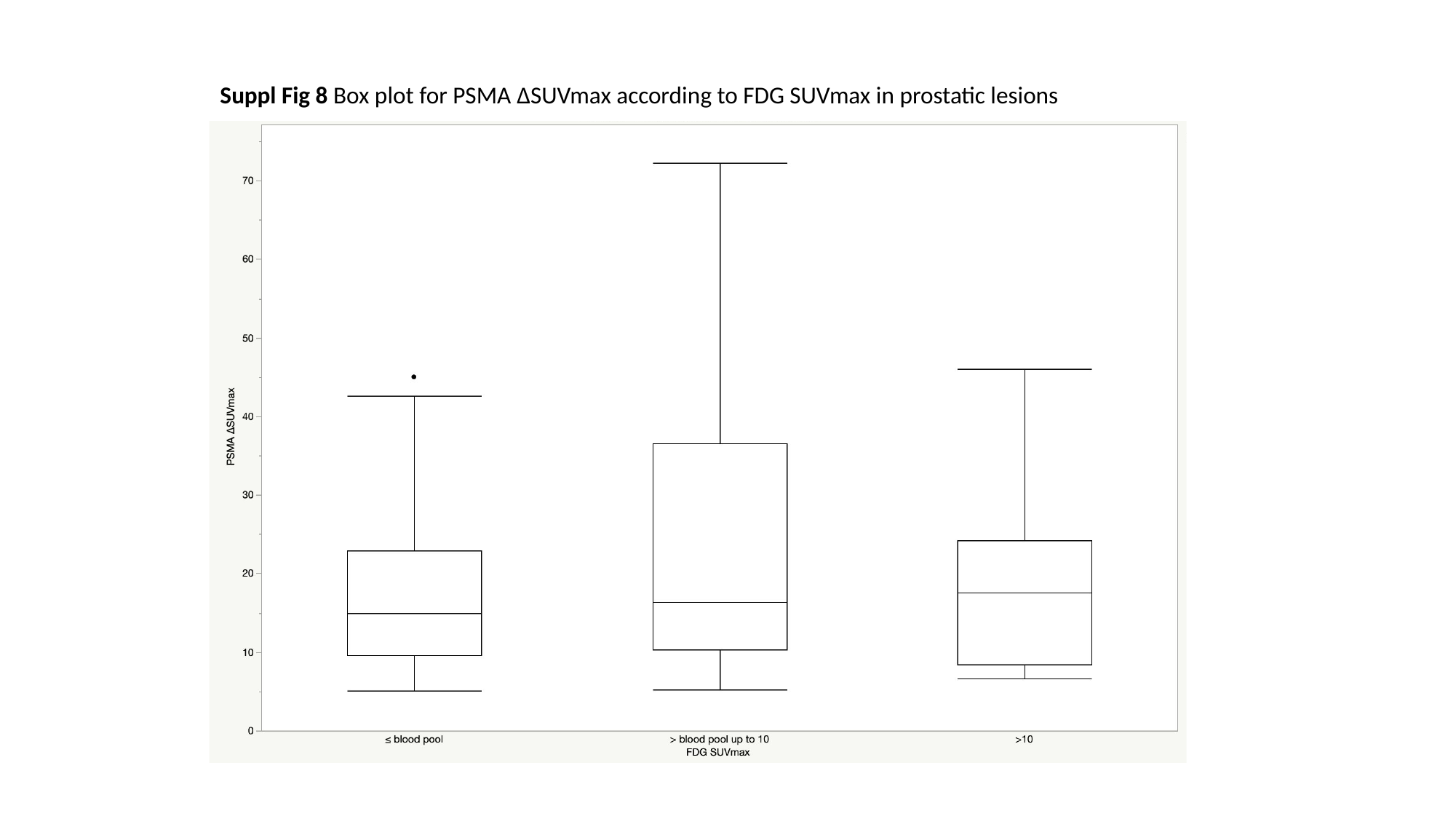

Suppl Fig 8 Box plot for PSMA ΔSUVmax according to FDG SUVmax in prostatic lesions
